# Supplementary material for: A set of multi-entry identification keys to African frugivorous flies (Diptera, Tephritidae)
Source: Zookeys. 2014 Jul 24;(428):97–108. doi: 10.3897/zookeys.428.7366 (PMC4143993; doi:10.3897/zookeys.428.7366)
Supplement: Supplementary material 6 — Key to Ceratitis [file zookeys-428-097-s006.zip › SF6_ZooKeys_key to Ceratitis/key/SF6_ZooKeys_key to Ceratitis/Media/Html/Ceratitis tripteris .htm]

Ceratitis (Pterandrus) tripteris (Munro)


***Ceratitis*** **(*Pterandrus*) *tripteris*
(Munro)**

*Pterandrus tripteris* Munro, 1957: 869.

Body length: 4.21 (3.95-4.45) mm; wing length: 4.55 (4.12-4.95) mm.

Male

Head: Antenna yellow. First flagellomere 2-3 times as long
as pedicel. Arista with short to moderately long rays; ventral rays shorter and
sparser than dorsal rays, especially basally. Frons convex; pale, center more
yellow; with short scattered setulae largely of same color as frons. Frontal
setae poorly developed. Face yellowish white; gena sometimes somewhat darker.
Genal seta and setulae dark, well developed. Cephalic setae usually all black,
occasionally few setae more reddish (perhaps discoloration?).

Thorax:
Postpronotal lobe yellowish white, without spot, at most with darker yellow
coloration near postpronotal seta. Scutal pattern: ground color grayish-brown,
dorsolaterally more yellow; with streaks and darker markings but without
distinct spots or clearly defined stripes, except prescutellar white separate
markings, with pale area in between. Scapular setae dark reddish or black.
Scutellum yellowish white, basally with two separate dark spots, apically with
three separate black spots, extending to basal 0.33. Anepisternum on ventral
half yellow; pilosity pale except few dark setulae along ventral margin ventral
to anepisternal seta.

Legs:
Yellow except where otherwise noted; setation typical for subgenus, mixed pale
and dark. Foreleg: coxa anteriorly brownish black; femur brown, pale along
ventral 0.33; at demarcation with row of longer setulae, basally pale, distally
dark; posteriorly with dorsal 0.33 brown, with bush of dispersed longer setulae
along entire length, posterodorsal row longer and dark, others pale; ventral
setae dark only on distal half; basally dispersed long setulae pale. Midleg:
femur anteriorly pale, ventral half brown except at apical margin where white,
ventrally with silvery shine when viewed from certain angle; with lump of dark
feathering in median part for 0.17 of entire length; tibia not broadened, pale
with brownish longitudinal streak; with black feathering dorsally with
dispersed row along distal 0.66 to 0.8 and ventrally with more appressed row
(as in other *Pterandrus* species) along entire length except basal and
distal end; second row of much shorter appressed feathering present posterior
of dorsal row for slightly more than distal half. Hindleg: femur yellow with
brownish streaks; with distinct black feathering on distal half dorsally and
and ventrally.

Wing:
banding yellowish brown. Marginal band forming continuous band with anterior
part of discal band; cubital band free; medial band absent; crossvein R-M just
beyond middle of discal cell. Apex of vein R_1_ distal to level of
crossvein R-M. Crossvein DM-Cu oblique anterobasally.

Abdomen:
Mostly yellow. Tergites 2 and 4 with pale gray band on posterior half. Tergite
3 with brown transverse band along� posterior half to 0.66. Male epandrium in
lateral view with posterior lobe of surstylus slightly curved and short,
anterior lobe well pronounced.

Female

As
male except for the following characters: Frontal setae well developed. Wing
banding slightly darker; marginal band partly interrupted but never fully. Legs
without feathering; mainly yellow; forefemur with ventral setae and
posterodorsal setulae dark; posteroventral setulae pale, at distal end few dark
setulae. Oviscape shorter than preabdomen. Aculeus at most six times longer
than wide; tip with distinct apical indentation and lateral margin slightly
sinuous.

(Description
after De Meyer & Freidberg, 2006)
